# Temporal precision and accuracy of audio-visual stimuli in mixed reality systems

**DOI:** 10.1371/journal.pone.0295817

**Published:** 2024-01-02

**Authors:** Daniel Eckhoff, Jan Schnupp, Alvaro Cassinelli

**Affiliations:** 1 School of Creative Media, City University of Hong Kong, Kowloon Tong, Hong Kong; 2 Department of Neuroscience, City University of Hong Kong, Kowloon Tong, Hong Kong; Panteion University of Social and Political Sciences, GREECE

## Abstract

Mixed Reality (MR) techniques, such as Virtual (VR) and Augmented Reality (AR), are gaining popularity as a new methodology for neuroscience and psychology research. In studies involving audiovisual stimuli, it is crucial to have MR systems that can deliver these bimodal stimuli with controlled timing between the onset of each modality. However, the extent to which modern MR setups can achieve the necessary precision and accuracy of audiovisual stimulus onset asynchronies (SOAs) remains largely unknown. The objective of this study is to systematically evaluate the lag and variability between the auditory and visual onset of audiovisual stimuli produced on popular modern MR head-mounted displays (HMDs) from Meta, Microsoft, HTC, and Varjo in conjunction with commonly used development environments such as Unity and the Unreal Engine. To accomplish this, we developed a low-cost measurement system that enabled us to measure the actual SOA and its associated jitter. Our findings revealed that certain MR systems exhibited significant SOAs, with one case averaging 156.63 ms, along with jitter of up to ±11.82 ms. Using our methodology, we successfully conducted experimental calibration of a headset, achieving SOAs of −3.89 ± 1.56 ms. This paper aims to raise awareness among neuroscience researchers regarding the limitations of MR systems in delivering audiovisual stimuli without prior calibration. Furthermore, we present cost-effective methods to calibrate these systems, thereby facilitating the replication of future results.

## 1 Introduction

Mixed Reality (MR) techniques, such as Virtual (VR) and Augmented Reality (AR), are gaining popularity as a new methodology for neuroscience and psychology research. Through visual, auditory, tactile, and other sensory modalities, MR can either substitute or augment the real world. VR, as defined by Milgram and Kishino, immerses participants in a virtual world, that can may mimic real-world environments or transcend physical reality by defying gravity, time, and material properties [1]. In contrast, AR augments the user’s perception of the real world by superimposing computer-generated sensory stimuli on top of their normal sensory input [2].

MR allows researchers to study complex and naturalistic behaviors in virtual or augmented environments while providing a very high level of experimental stimulus control. Some neuroscience or psychology studies require the fabrication of complex custom setups for stimulus presentation, which can be much easier to create in MR. MR setups often make it easy to provide rich multisensory stimulation, enhancing the ecological validity of experiments because it facilitates the delivery of stimuli and sensory contexts that more closely resemble real life, potentially leading to more generalizable and valid explanations of cognitive processes. In that sense, Rizzo et al. referred to VR environments as the “ultimate Skinner box” [3]. Furthermore, it has been shown that real-life and VR scenarios can be indistinguishable on a psychophysiological level, which sets them apart from conventional 2D laboratory settings [4].

The availability of affordable head-mounted displays (HMDs), and the relatively easy development of complex MR experiences through MR development environments such as Unity or Unreal make MR more accessible to researchers. We expect this to have a major impact on cognitive neuroscience research. Newer headsets, such as the Meta Quest Pro, the Varjo XR-3, and the Apple Vision Pro, also have eye-tracking systems built into the headsets, providing another useful feature that can facilitate the collection of data of interest to neuroscientists and cognitive and sensory psychologists. It is also expected that future headsets will incorporate additional sensors that can be useful for neuroscience studies. For example, there are already HMDs that integrate electroencephalography (EEG), electromyography (EMG), electrodermal activity (EDA), photoplethysmography (PPG), and eye tracking sensors in a single headset, such as the OpenBCI Galea.

Due to the increasing use of MR in neuroscience and psychology research, companies specializing in neuroimaging and physiological equipment, such as ANT Neuro or BIOPAC, are releasing products specifically designed for the use with HMDs. We therefore expect MR to have a significant impact on visual, auditory and, in due course, tactile, perceptual and cross-modal studies in psychology and neuroscience.

### 1.1 Why timing is important

In psychology or neuroscience experiments, it is often of paramount importance to present visual and auditory stimuli to participants with high precision, accuracy, and temporal synchrony. Stimulus onset asynchronies (SOAs) should be tightly controlled and their timing very consistent, and any variability or inaccuracy in stimulus timing must be quantified. Unknown inaccuracies in equipment might lead to unreprodudible results, as highlighted in recent discussions regarding the *replication crisis* [5]. For example, a recent study using audiovisual stimuli to study speech perception was retracted due to synchronization issues of the audiovisual stimuli [6].

In many experiments, the visual and auditory stimuli are presented together to be perceived as a single multisensory event or object, and poor synchronization can have adverse effects on the desired cross-modal binding. While some experiments will simply want to ensure reliable and accurate synchrony, others may even intentionally introduce known delays of one stimulus modality relative to the other to systematically explore the effects of SOA.

The audio-visual temporal binding window (AV-TBW) refers to the range of SOAs over which there is a very high probability that the auditory and visual stimuli are bound, that is, perceived as a single bimodal stimulus. As the asynchrony between the auditory and visual stimuli increases, the likelihood of a single, bound percept decreases. In contrast, the intersensory temporal contiguity window (ICTW) is defined as the minimum auditory/visual onset aynchrony that leads to a reliable perception of two separate unimodal stimulus events rather than a single multimodal one [7]. The ICTW can vary depending on stimulus type, context and participant. ICTWs as low as 20 ms have been reported by Hirsh and Sherrick [8], but ICTW values of around 60 ms [9] are perhaps more typical, and certain types of complex stimuli, such as speech, appear to be less sensitive to temporal discrepancies, with ICTW reported to be around 180 − 240 ms [10–12].

Research into sensory psychology and neuroscience often requires very precise measurements of the temporal relationships between stimulus delivery and various response measures, including reaction time measurements or measurements of physiological responses, including autonomic responses such as pupil dilation or galvanic skin responses, to electrophysiological measures such as event-related potentials (ERPs) or time-frequency responses (TFRs) recorded via EEG. To permit such studies, being able to determine and control stimulus onset times with precision is of obvious importance. Reaction time measures and ERPs in particular require high temporal precision.

ERPs are the result of averaging time-locked electrical changes in brain activity that occur in response to specific stimuli over many trials. This technique allows for the precise measurement of the brain’s response to specific stimuli, providing insights into the neural mechanisms that underlie various cognitive processes. By examining these ERPs, researchers can determine the timing and nature of the brain’s processing of sensory information from different modalities.

Typically, ERP recordings have a duration of a few hundred milliseconds, and researchers may be interested in documenting temporal features of ERP wave forms with a temporal resolution of a few milliseconds. Unknown lags or temporal jitter of only a few tens of milliseconds in audiovisual stimulation could therefore reduce the quality of ERP data or make them harder to interpret.

The effects of SOAs on multimodal integration of audiovisual stimuli on ERPs have been shown to occur as early as 40 ms after stimulus onset [13]. Kaya and Kafaligonul demonstrated that visually evoked stimuli modulate the N1 and P1 components based on the asynchrony with an auditory stimulus, up to 40 ms [14]. Moreover, the P1 was modulated when the auditory stimulus appeared before the visual stimulus, while the N1 was modulated when the auditory stimulus appeared after the visual stimulus. Similarly, work by Ren et al. showed that audiovisual integration in ERPs occurs only within a SOA range of −50 to 50 ms [15].

### 1.2 The importance of accurate event markers

To make the accurate interpretation of neuroimaging data possible, a low temporal jitter of stimulus onsets, as well as accurate timestamps marking these onsets (also referred to as event markers), are required. Even quite modest inaccuracies in the timing of event markers or stimulus onsets can significantly impact the quality of neuroimaging results. For instance, a jitter of 10 ms has been shown to induce significant effects on the P1 and N1 components of ERPs [16].

Many EEG experimental setups make use of Serial Communication protocols to provide event markers that are later used to extract temporal epochs from the recorded EEG signals and calculate averaged ERPs. However, that protocol also has been shown to be able to induce significant delays and jitter that can affect the quality of ERP recordings [17, 18]. An alternative method consists in saving log files with timestamps generated by the stimulus presentation system. This approach, however, still requires some mechanism for clock synchronization between the device presenting the stimuli and the device recording the EEG data respectively, and this can be challenging to achieve when millisecond accuracy is necessary.

One potential issue with procedures that rely on collecting time stamps during the experiment is that the software collecting the time stamps will likely have easy access only to the time on the system clock when the presentation software decides to initiate stimulus presentation, not the time when the stimuli actually start. The time lag between a start command and actual stimulus onset is often very small, but one cannot always safely assume that it will be negligible. Given the inherent complexity and independence of contemporary multi-layer and multi-threaded graphics and audio pipelines which run on non-real-time operating systems, the time it takes for stimuli to finally reach the output stage can vary somewhat from trial to trial and may be difficult to estimate accurately a priori.

### 1.3 Temporal precision and accuracy in common equipment and mixed reality systems

In the fields of experimental psychology and sensory neuroscience, it is common practice to characterize the precision and accuracy of common equipment used for stimulus delivery empirically [19–22]. In contrast, so far there has been only a small amount of research to assess the suitability of current consumer-grade MR systems and software frameworks for use in scientific research involving precise and accurate timing of stimuli. Recently, Wiesing et al. investigated the precision and accuracy of timing mechanisms of the Unreal Engine 4 and SteamVR with the HTC Vive VR system in neuroscientific research [23]. Their results showed that, while stimulus duration was highly accurate, built-in timing procedures suffered from variable reaction times and inaccurate stimulus onsets. In another study, Tachibana and Matsumiya evaluated the accuracy and precision of visual and auditory stimuli generated in VR head-mounted displays (HMDs) from HTC and Oculus using Vizard on both Python 2 and 3 [24]. They found an 18 ms time lag for visual stimuli and a 40 − 60 ms time lag for auditory stimuli, with low jitter in the order of 1 ms for visual and 4 ms for auditory. The results were consistent in both Python 2 and 3.

### 1.4 Research problems and aims

**Fig 1:**
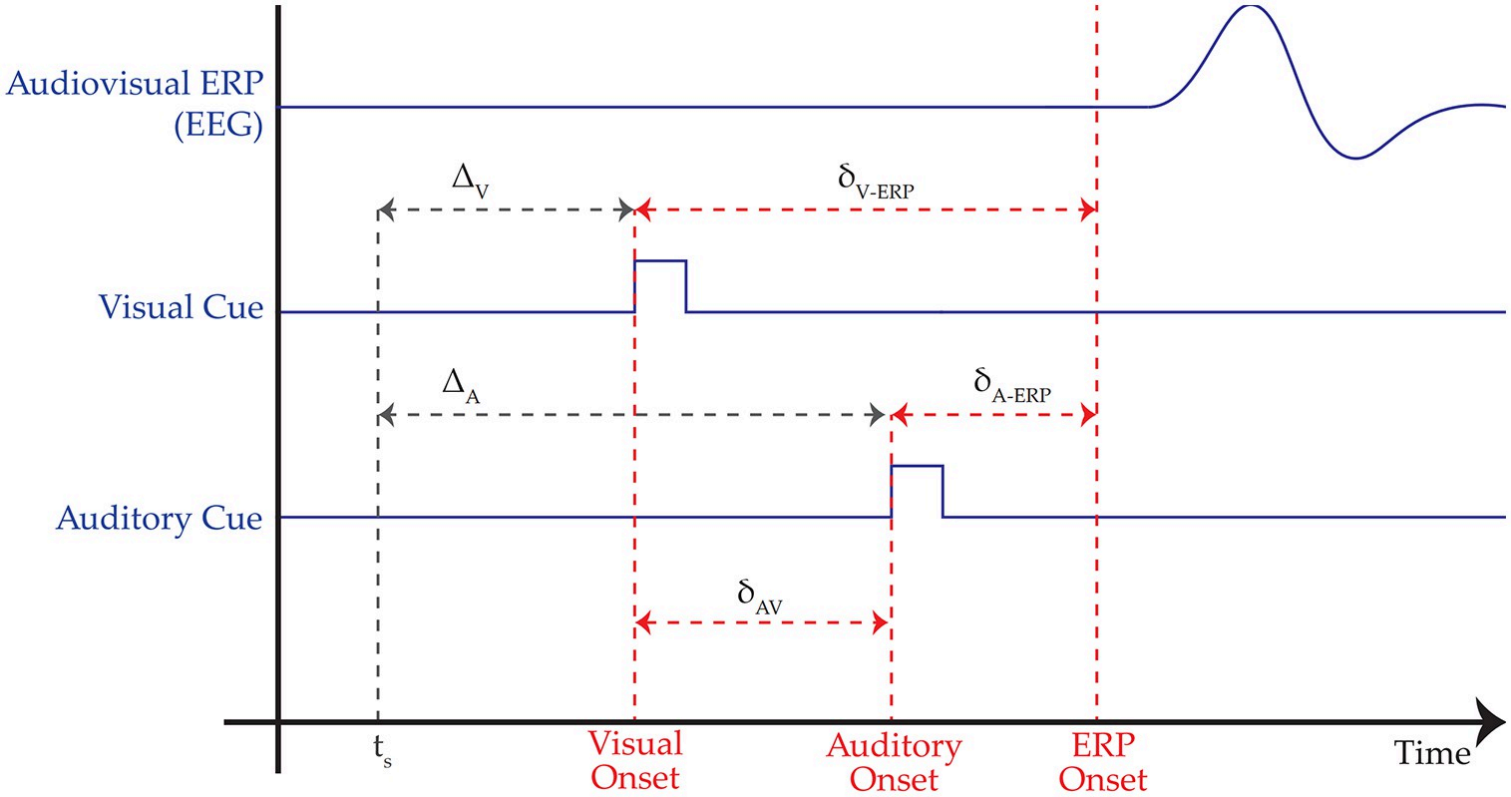
Problem illustration. The processing delays in the audio and video pipelines can be variable and are not known precisely, which makes it difficult to estimate the auditory delay (*Δ*_*A*_ and the visual delay *Δ*_*V*_, as well their jitter). Fortunately, the unimodal delays between the time when the stimulus software decides to trigger each stimulus and the stimulus actually appearing are often inconsequential, as long as the difference in auditory and visual onsets (*δ*_*AV*_) is known. Our device measures these delays very precisely. With this knowledge, it is possible to ensure control over the onset asynchronies (*δ*_*AV*_) through complementary delays implemented in the stimulus software.

In Fig 1, the problems of temporal precision and accuracy on MR systems are displayed in a schematic time plot. The audio-visual processing pipeline for AR and VR systems are complex and composed of multiple layers of proprietary hardware and software. This leads to inevitable delays and jitter between graphics and audio output, which cannot be estimated a priori based solely on the system architecture. The time when a start command for a stimulus is issued (*t*_*s*_) may not serve as a very reliable time stamp or event markers for calculating ERPs, given the complexity and multi-threaded nature of the audio and graphics processing pipelines.

Both the video and auditory signals have their own specific delays and jitter (respectively *Δ*_*V*_ and *Δ*_*A*_ in Fig 1). Stimuli in each modality evoke time-locked brain activity associated with auditory and visual sensory processing respectively, as well as brain activity related to multisensory integration [13]. Delays in these signals will result in modulated peaks of the ERPs associated with their processing, making comparisons to related work challenging.

To date, there has been no systematic study quantifying the temporal lag and jitter between visual and auditory stimuli in consumer and professional MR hardware used with development environments such as Unity and Unreal. In this study, we compare the accuracy and precision of audiovisual stimuli onsets in several commonly used development environments for MR applications and HMDs. Our objectives are to: **(1)** determine the range of timing performance across platforms and headsets; **(2)** present an easy and inexpensive method for measuring the timing of visual and auditory stimuli with sub-millisecond accuracy and precision; **(3)** assess the technical capability of MR systems to achieve sufficient timing for cross-modal neuroscience or behavioral studies.

## 2 Methodology

### 2.1 Head-mounted displays

We measured the stimulus onsets asynchronies of audiovisual stimuli using the following headsets: Meta Quest 2, Meta Quest Pro (Meta Platforms, Inc., Menlo Park, California, United States), HTC Vive Pro (HTC Corporation, Xindian, New Taipei, Taiwan), and Varjo XR-3 (Varjo Technologies Oy, Helsinki, Finland). Detailed specifications of each headset are presented in Table 1. We tested the SOAs for auditory stimuli presented through both integrated speakers and external headphones for Meta Quest 2, Quest Pro, and HTC Vive Pro. However, due to its lack of an integrated 3.5mm jack, we only measured audiovisual synchronization using the integrated speakers of the Microsoft HoloLens 2. Additionally, the Varjo XR-3 does not have built-in speakers, so only external wired-headphones were used to evaluate audiovisual synchronization performance. We employed Sennheiser HD-280 Pro headphones (Sennheiser electronic GmbH & Co. KG, Wedemark-Wennebostel, Germany) connected to the headsets via a 3.5mm jack.

**Table 1:** Comparison of key specifications for the tested head-mounted displays.

Headset	Stand-alone	Headset Type	Display Type	Refresh Rate	Resolution per eye	FoV	Sound	Supported Platforms
**Meta Quest 2**	Yes	AR/VR	LCD	60,120 Hz	1832 × 1920	89°	2 speakers 3.5mm	Oculus Mobile
								OpenVR
								OpenXR
**Meta Quest Pro**	Yes	AR/VR	LCD	72,90 Hz	1800 × 1920	106°	2 speakers 3.5mm	Oculus Mobile
								OpenVR
								OpenXR
**Microsoft HoloLens 2**	Yes	AR	Waveguide	75 Hz	1440 × 936	43°	2 speakers	WindowsMR
								OpenVR
								OpenXR
**HTC Vive Pro**	No	AR/VR	AMOLED	90 Hz	1440 × 1600	110°	2 speakers 3.5mm	OpenVR
								OpenXR
**Varjo XR-3**	No	AR/VR	uOLED LCD	90 Hz	1920 × 1920 2880 × 2720	115°	3.5mm	OpenVR
								OpenXR

The standalone headsets were updated to the most up-to date version of their operating systems at the time of the data collection (December, 2022). The Meta Quest 2 and Pro were tested using the latest Meta Quest software version 47. We set the display refresh rate to the highest possible, which is 120 Hz for the Meta Quest 2 and 90 Hz for the Meta Quest Pro. We tested The Microsoft HoloLens 2 using the latest version of the Windows Holographic, version 22H2, build number 20348.1528. The tethered headsets, HTC Vive Pro and the Varjo XR-3, were driven by a PC containing an Intel i9-9900k, 32GB 3200 MHz DDR4 RAM on a motherboard based on the Z390 chipset. The chipset includes a Realtek S1220A for delivering sound. We used an nVidia GeForce RTX 2080Ti as the GPU to drive both the Vajro XR-3 and the HTC Vive Pro.

### 2.2 Software

We sought to compare the audiovisual stimuli onset asynchronies and their jitter with the two most commonly used MR development environments: Unity (2021 LTS) (Unity Software Inc., San Francisco, California, United States) Unreal Engine (4.27) (Epic Games, Inc., Cary, North Carolina, United States). Our benchmark application displays a white (RGB: 255, 255, 255) rectangle on a black (RGB: 0, 0, 0) background and plays a sine wave, both for 500 ms.

We created the benchmark testing application in the manner that might be expected from a normal user of the engine and the necessary software plugins. We followed the provided documentation and followed official recommendations to reduce audio latencies and jitter, such as choosing the smallest buffer sizes for audio. For Unreal, we set the option *Callback Buffer Size* to 512 and *Number of Buffers to Enqueue* to 2. For Unity we set the option *DSP Buffer Size* to *Best Latency*. We excluded any other advanced and undocumented tweaks.

### 2.3 Measurement device

As previously discussed, it is not easily possible to accurately determine the actual onset time of a visual or auditory stimulus from when the software initiates a *start* command. Therefore, the most dependable approach to measure the SOAa is by assessing their relative onsets.

**Fig 2:**
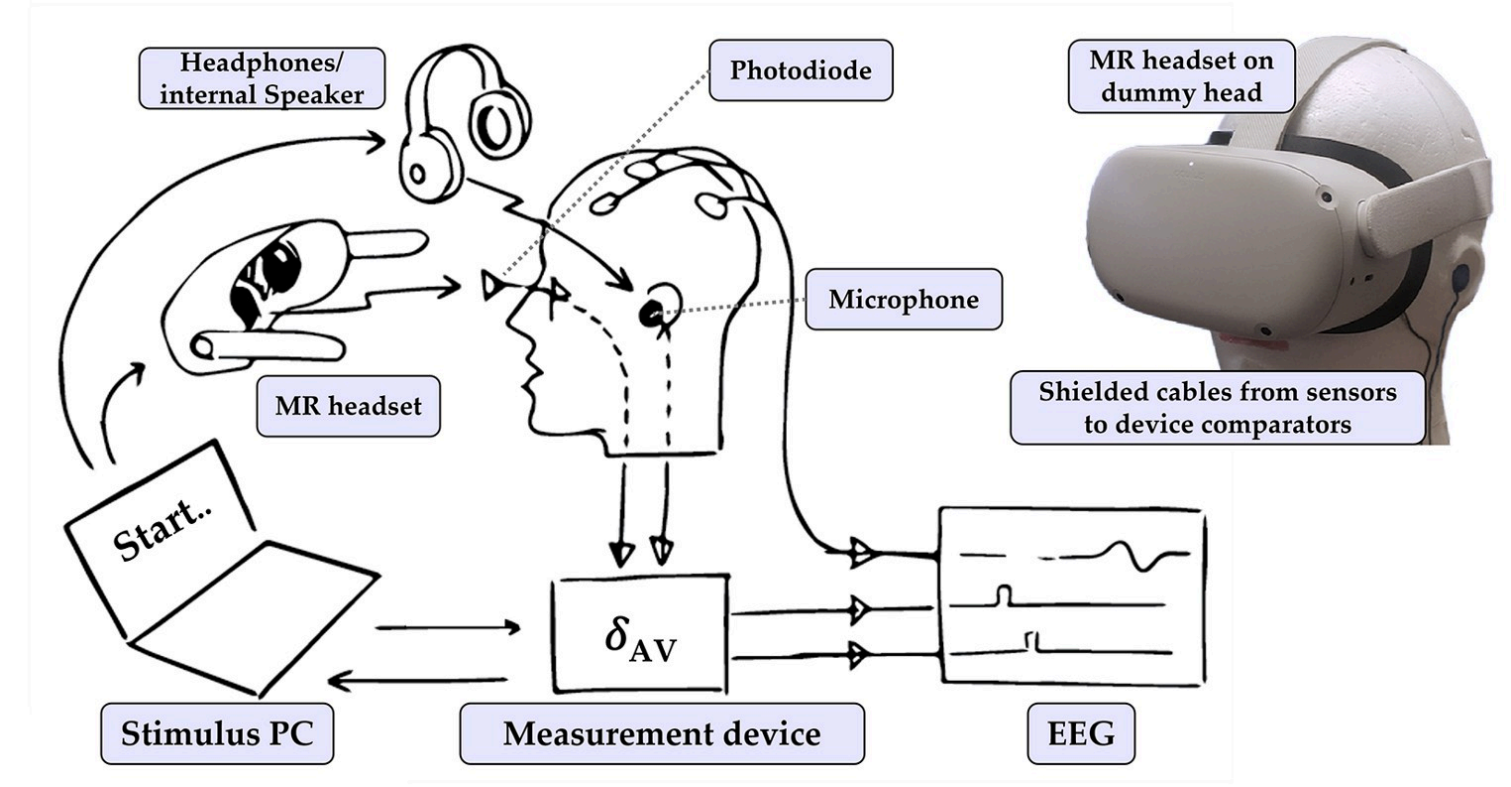
Schematic illustration of our measurement setup. Our measurement device is capable of detecting auditory and visual stimulus onsets, their asynchrony *δ*_*AV*_ and jitter. The stimuli are generated by a stimulus computer or a standalone HMD. The audio and video signals are detected by specialized sensors. SOAs and their jitter can be determined offline using a dummy head for calibration purposes. Alternatively, our device can be used during an experiment to generate real-time TTL signals, that serve as precise event markers of the stimulus onsets for each modality.

**Fig 3:**
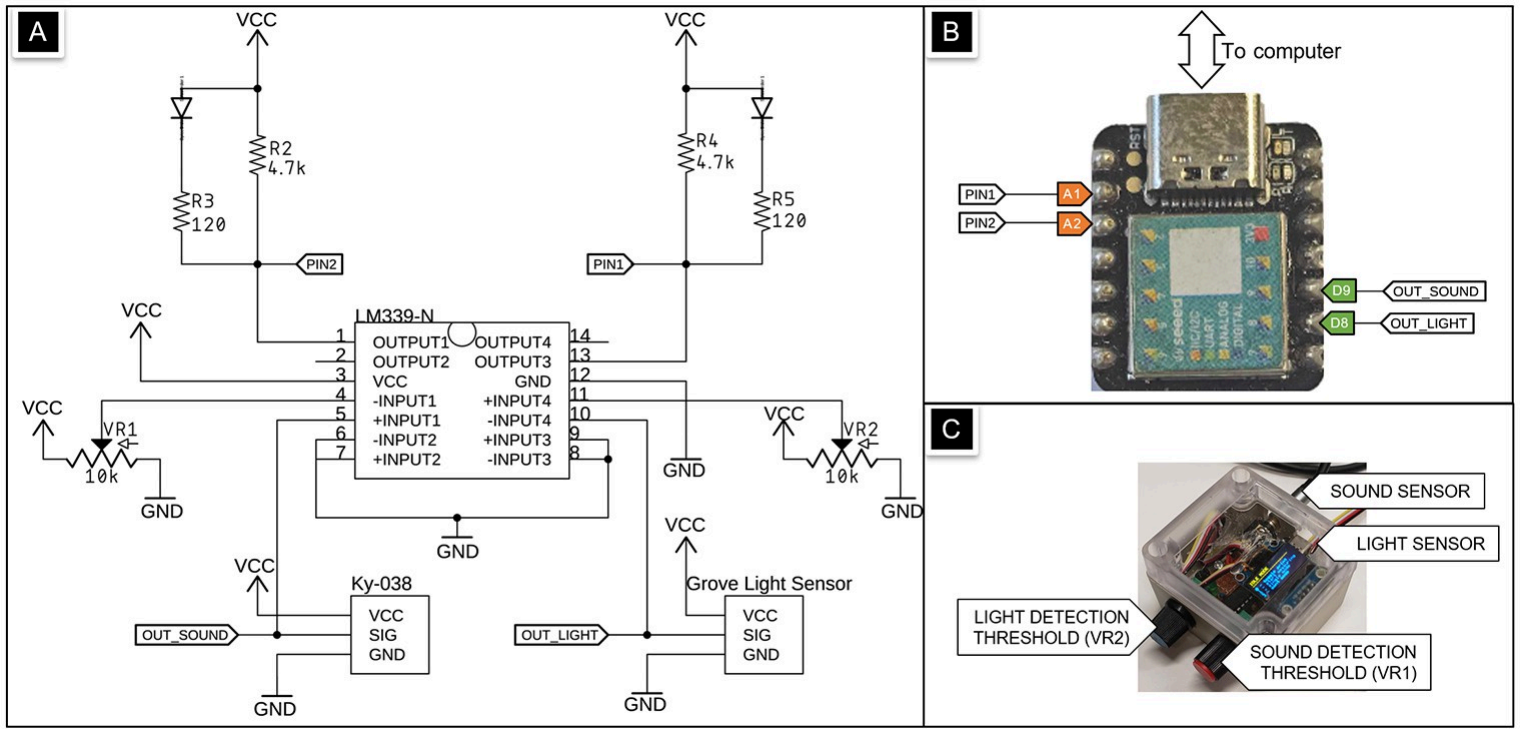
Schematic illustration of our measurement device. (A) Schematic diagram of our real-time device for measuring auditory and visual stimulus onset times. The circuit consists of a light sensor and a microphone, which generate analog signals that are conditioned and compared against adjustable thresholds (VR1, VR2) using an operational amplifier comparator (LM339N). (B) The LM339N can trigger programmable interrupts on the Seeeduino Xiao through the pins D8 and D9. The device can communicate with a computer via a USB-Serial modem for data sending and command input. The device also produces two TTL outputs (OUT_SOUND, OUT_LIGHT) for exact onset of the visual and auditory stimuli, which can be used as event markers. (C) Photograph of our measurement device.

To measure auditory and visual stimulus onset times, we built a dummy head with integrated microphones in place of the ears and light sensors over the eyes. The experimental setup is illustrated in Fig 2, while the detailed circuitry and measurement apparatus are depicted in Fig 3. Both the microphone and the light sensor can also be attached directly to the HMD’s speaker and display respectively, the later thanks to a small suction cup. This may be necessary when the environment is noisy or when the headset intermittently flashes an infrared beacon in order to detect the presence of the user or to perform eye tracking. This feature is commonly observed in modern devices such as the Meta Quest Pro and the Varjo XR-3 which were employed in our study.

To register the auditory and visual stimulus onsets with millisecond or even sub-millisecond accuracy, a device is required that does not introduce unknown delays. Therefore, the sensors cannot be simply connected to a conventional computer running a multi-threaded operating system, rather than a real-time operating system. The problem becomes even more acute when signals are monitored through devices that are connected to conventional USB ports, as these can introduce additional latency and jitter.

Microcontrollers are designed specifically for such time critical measurements as they can run bare-metal and unthreaded software. In our device, we used the Seeeduino Xiao SAMD21 development board relying on the Microchip SAMD21G18 ARM CortexM0, a 32-bit microcontroller running at 48MHz.

We used external hardware interrupts for measuring the stimulus delays. The SAMD21 provides programmable interrupts (raising, falling or high/low) on 16 digital pins, each capable of triggering an interrupt service routine (ISR) with a worst case latency of seven clock cycles—or about 145 nanoseconds. Since the interrupt pins need TTL signal, we used a LM339 quad-comparator IC, with thresholds that can be adjusted manually through a pair of linear potentiometers (one for the audio signal and another for the light sensor). The Grove Light sensor’s photodiode and the Keyes KY-O38 electret microphone signals are filtered and pre-amplified before reaching the comparator inputs through a shielded cable (see Fig 3).

We want our device to be capable of communicating with a computer via an USB-Serial modem, not in order to signal the onset of the auditory or visual stimulus, but to retrieve the measured delays or their average over many trials and store them for further processing, or to issue commands or set operational parameters (calibration of thresholds for detection, number of measurement cycles to perform, stop or resume operation, etc).

The computer can issue a command requesting a single measure. This puts the device in listening mode, and after detecting both onset times for audio and video, it computes the lag between them and sends this value back to the computer through the USB connection. Another command can put the device in free-running mode, passing also the number of measurements the device should gather before transmitting the average delay and jitter (as the standard deviation from the sample mean). The user can collect the final results (average and standard deviations) or can retrieve each measure separately for further analysis on the computer. In either case, the device returns back to IDLE mode when measurement is completed, until a new command to collect further measurements is received.

Below we report results obtained from the use of this device to precisely characterise the audio-visual delay of several MR headsets. This enables us to compensate the delay by software if desired. The device also produces two TTL outputs, each indicating the exact onset of the auditory and visual stimuli as these could be fed directly to the EEG recording computer or amplifier as additional traces registered without delay (no transmission protocol, just electrical signals).

## 3 Results

**Fig 4:**
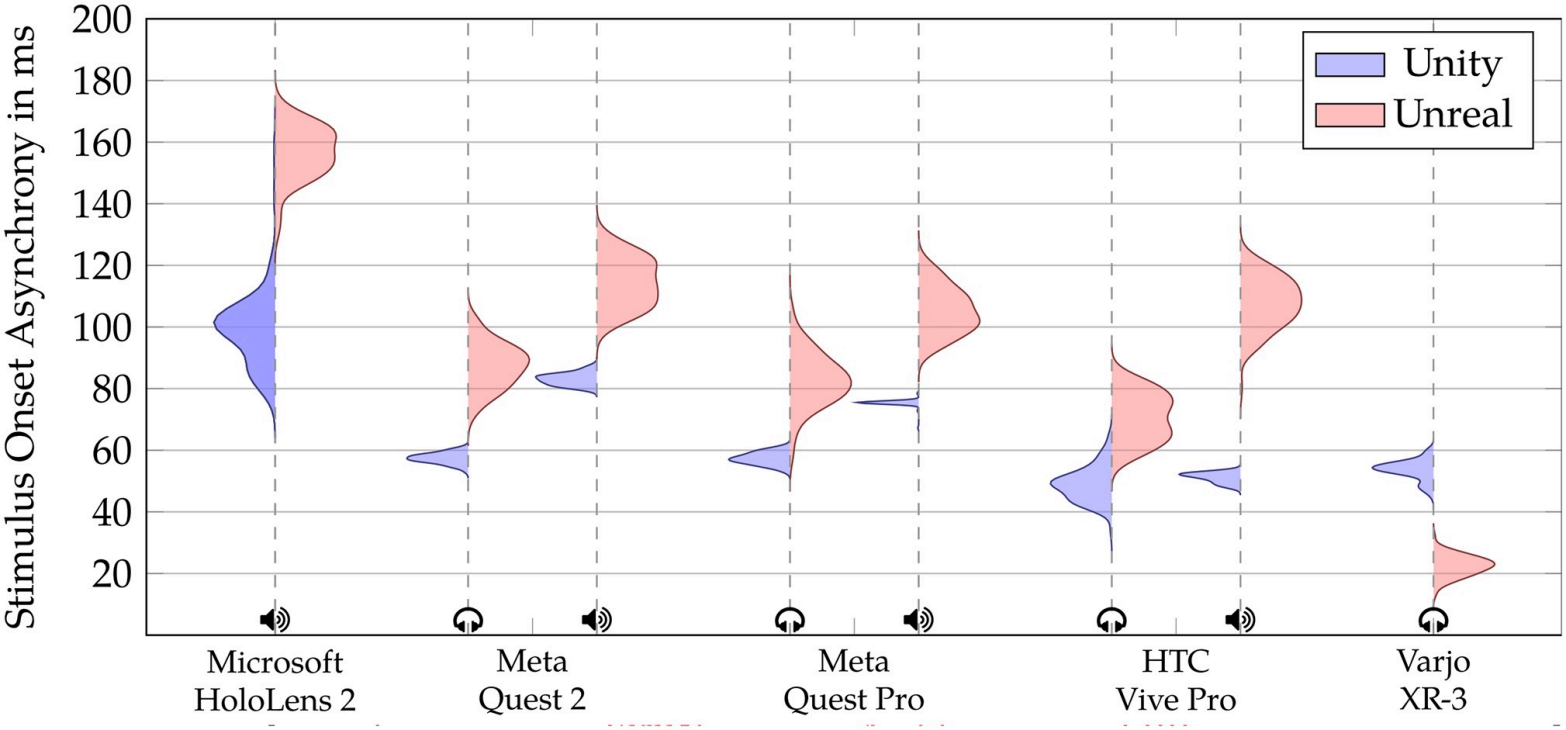
SOA distribution across MR setups. Violin plots depicting the distribution of SOAs for each Head-Mounted Display tested with Unity (Blue, on the left) and Unreal (Red, on the right), along with the type of speaker used: headphones (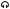) and internal speaker (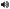).

Table 2 and Fig 4 display the descriptive results and the data distribution for the evaluated MR systems, which include the Meta Quest 2, Meta Quest Pro, Microsoft Hololens 2, HTC Vive Pro, and Varjo XR-3. The assessment of audiovisual synchronization performance was based on both mean and standard deviation lag values calculated from 100 audiovisual stimulus presentations.

**Table 2:** Stimulus Onset Asynchrony (SOA) summaries by Head-Mounted Display (HMD), operating systems, sound source and engine.

HMD	OS	Sound Source	Engine	SOA in ms	
				Mean	SD
Meta Quest 2	Android	Internal Speaker	Unity	83.05	1.90
			Unreal	114.48	7.17
		Headphones	Unity	57.42	1.70
			Unreal	87.41	7.17
Meta Quest Pro	Android	Internal Speaker	Unity	75.41	1.03
			Unreal	87.82	6.19
		Headphones	Unity	57.31	1.95
			Unreal	82.82	8.67
Microsoft HoloLens 2	Windows 10	Internal Speaker	Unity	99.09	11.82
			Unreal	156.63	8.44
HTC Vive Pro	Windows 11	Internal Speaker	Unity	48.57	5.48
			Unreal	70.77	7.66
		Headphones	Unity	51.12	1.60
			Unreal	107.85	8.18
Varjo XR-3	Windows 11	Headphones	Unity	53.49	3.15
			Unreal	22.46	3.33

We found that SOAs can vary greatly depending on the development environment used (Unity, Unreal Engine), the sound source (headphones, internal speaker), and the HMD. Even for the same HMD, SOAs can vary depending on the development environment and sound source.

Overall, the Varjo XR-3 running on Windows 11 with external headphones achieved the lowest SOA, with a measurement of 22.46 ms and a jitter of 3.33 ms. The standalone devices Meta Quest 2 and Pro achieved the lowest jitter when using Unity, with 1.70 ms for the Meta Quest 2 and 1.03 ms for the Meta Quest Pro.

To investigate the feasibility of achieving adequate control over the SOAs, we experimentally calibrated the Unity application for the Varjo XR-3. We delayed the function responsible for displaying the visual stimuli by five frames (54 ms, assuming a constant frame time of 11.11 ms and display refresh rate of 90 Hz) in reference to the function to play an auditory stimulus within the Unity application. This resulted in a reduced SOA and jitter from 53.49 ± 3.15 to −3.89 ± 1.56 ms. The negative value indicates that the auditory stimulus was detected prior to the visual stimulus.

## 4 Discussion

This study systematically evaluated the accuracy and precision of audio–visual stimuli presentations in Mixed Reality headsets. As expected, the results show that the audiovisual synchronization performance of MR systems can vary significantly depending on the combination of headset, operating system, and development environment. Our study highlights the importance of following proper characterization procedures before embarking on time-sensitive experiments. It is important to note that the values reported here, or those from other researchers, can only highlight this problem and should not be used as absolute references. The precise values may vary as a function of differences in the experimental setup or vary from unit to unit, even for the same HMD model, particularly if HMDs run different firmware versions. However, we have designed our own cost-effective and easily replicable measurement device.

To accurately measure SOA down to the millisecond or sub-millisecond level, a device that doesn’t introduce unknown delays is needed. This rules out using sensors connected to a conventional computer with a multi-threaded operating system, especially through USB ports, which can add latency and jitter. We used an external microcontroller running in real time to detect the onsets of auditory and visual stimuli and buffers the event times in the microcontroller’s memory until the host computer is ready to collect them. Thus, the accuracy of the measurements is limited only by the sample rate of the microcontroller and independent of the workload on the host computer.

Knowing the specific SOAs for a given setup, the system can be easily calibrated through software time compensation. Using time compensation by software, we successfully reduced the audio-visual delay in the Varjo XR-3, achieving a SOA of −3.89 ± 1.56 ms. This is an acceptable audiovisual lag as it is not noticeable to participants [8]. A jitter as low as the one reported will have a negligible effect on the outcomes of experiments involving neuroimaging, such as EEG [16]. It is well within the range of other well-established stimulus presentation frameworks such as PsychoPy, as well as standard 2D lab environments with a low-latency LCD monitor.

We assessed the SOAs using both the internal speakers of the MR headsets and external headphones connected to either the standalone HMD (Meta Quest 2, Meta Quest Pro) or the PC running the application for delivering the audiovisual stimuli. We observed a lower lag, but comparable jitter, when headphones were used for the Meta Quest 2 and Quest Pro. This could be due to additional post-processing that Meta applies when the sound is played through their internal speakers. In contrast, for the HTC Vive Pro, we observed the opposite effect with the Unreal-built application. Using external headphones instead of the built-in speakers resulted in an unexpected additional 37 ms of SOA, revealing unknown differences in the audio processing between SteamVR and Windows 11 depending on whether external headphones or integrated speakers are used.

We also found that applications built with Unreal-engine lead to a higher jitter for the Meta Quest 2, Meta Quest Pro, as well as the Vive Pro of up to 8.67 ms. The Microsoft HoloLens 2 had a high jitter of 11.82 ms for the Unity and 8.44 ms for the Unreal application. As pointed out by Williams et al., a jitter of 10 ms on stimulus onsets/event markers can already impact significantly on the amplitudes of the ERP components [16]. As the jitter can not be simply corrected for, we recommend that developers take into account the potential impact of hardware and engine choice on the accuracy of audiovisual stimuli in their applications and statistical analysis, particularly when they involve EEG recordings.

### 4.1 General recommendations

In general, researchers should prioritize low jitter (precision) over low delay (accuracy) when selecting and configuring the hardware and software for their experiments. A delay can be easily compensated by introducing an ad hoc delay, as shown in our experiment. High jitter, on the other hand, cannot be easily corrected.

We have observed that the choice of development environment, HMD, and audio output device (headphones or speakers) can have a significant impact on SOAs. However, we cannot recommend a specific combination due to the significant variation observed between HMDs, development environments, and even the type of speakers used. Furthermore, we anticipate that these variations may significantly differ depending on the development environment version, operating system, firmware of the headset, and audio and video driver versions among others [25].

Instead, this study emphasizes the importance of thoroughly measuring and calibrating the system to ensure accurate and reproducible results before performing any experiments. To achieve low delay and, more importantly, low onset variability prior to calibration, we recommend the following general methods:

When it comes to the auditory stimuli, the simplest approach to reducing delay and onset variability is to select the smallest audio buffer size supported by the hardware and software. However, this strategy comes at the expense of CPU performance and increases the likelihood of buffer underruns. A buffer underrun occurs when the amount of data stored in the buffer is insufficient to meet playback demand, resulting in missed stimuli, audio stuttering, and distortion. In addition to opting for the smallest available buffer size, the use of low overhead drivers such as the Audio Stream Input/Output (ASIO) driver protocol can help reduce the delay and variability of auditory stimuli onsets [25]. It is worth noting that certain built-in sound cards, such as those commonly found in notebooks and motherboards, may not support these drivers. External sound cards typically support ASIO or other low overhead protocols. However, there may be situations where using an external sound card is not feasible. For instance, standalone HMDs like the Microsoft HoloLens 2 or Meta Quest 2 might not support the use of an external sound card.

Regarding the visual stimulus, it is crucial for researchers to ensure that their experimental software maintains a consistent frame rate, ideally matching the maximum refresh rate of the display. Fluctuations in frame rate can have a significant impact on the onset and duration of the visual stimulus. By achieving a stable frame rate, researchers can lower the jitter of the onset and duration of visual stimuli. Additionally, the ability to control the SOA by delaying the visual stimuli by a specific number of frames is only feasible when the frame rate remains stable. Moreover, the choice of display technology in HMDs can also influence the onset and variability of visual stimuli. Different display technologies, such as LCDs and OLEDs, exhibit distinct temporal resolutions when presenting various transition times (e.g., gray-to-gray, black-to-white). For example, a black-to-white transition may occur faster than a 10% to 90% gray transition [26, 27]. Some HMDs, like the Varjo XR-3, even integrate both LCD and OLED panels within a single display. This HMD utilizes an OLED display for central vision and an LCD for peripheral vision, potentially introducing variations in SOAs depending on the stimulus location on the display.

Furthermore, when very short auditory and visual stimuli are presented, it is essential for researchers to ensure that their system is capable of delivering such short stimuli. For example, Tachibana and Matsumiya demonstrated that certain systems may not be able to play auditory and visual stimuli with a duration of 22.22 ms or shorter at all [24]. The accuracy of such short stimuli depends on the frame rate and display refresh rate set by the running software program. Increasing the frame rate will increase accuracy, and the stimulus duration should be a multiple of the frame rate interval. For example, traditional computer monitors run at 60 Hz, equivalent to 16.67 ms per frame, which is clearly too slow to implement visual stimuli of 10 ms duration. Modern HMDs often run at 90 Hz or faster, but there is no accepted standard, and performance for very short stimuli is obviously greatly affected by this parameter. As discussed by Tachibana and Matsumiya [24], the Meta Quest 2 and Pro, as well as some other HMDs, may use a technique called *Asynchronous SpaceWarp* within the video pipeline, which technically halves the framerate of the application in favor of improved performance and reduced motion sickness. Because researchers are sometimes unaware of such details, they should verify the actual frame rate of their experimental software and display refresh rate used in their experiments.

Before compensating for delay, the stimulus-presenting computer (or standalone HMD) should be configured to prevent automatic updates. Operating systems, audio and video drivers may revert to previous configurations, install different drivers, firmwares, or update parts of the system without the user’s knowledge, potentially affecting the delay and jitter of auditory and visual stimuli and rendering previous measurements and calibrations useless [25].

Once these software and hardware optimizations are in place, accurate measurements of jitter and delay can be done following the procedures described earlier in this article. Armed with this information, the experimenter can compensate the delay by software and/or include this error in subsequent statistical analysis.

### 4.2 Limitations

One limitation of our study is that we only tested applications built with Unity or Unreal Engine. While native applications written for a specific hardware platform may achieve better results and/or offer more customization to the audio and graphics pipelines, we focused on these two engines as they are very popular in both industrial and scientific settings. Chénéchal and Chatel-Goldman, as well as Tachibana et al. recommended using Python for VR studies [24, 28]. It’s worth noting that these Python packages do not support all HMD hardware or the most popular MR software frameworks.

Another limitation is that we used rather simple stimuli. The complexity of the stimuli may also affect the onset of visual stimuli. For example, more complex visual stimuli, such as text, 3D objects, or the use of advanced rendering techniques, can greatly affect the onset of a stimulus. Also, the use of spatialized sounds requires additional post-processing that can affect the time it takes for the stimuli to play.

Some neuroscience studies use auditory and visual stimuli that are significantly shorter than the stimuli used in the current study. As a result, another limitation of this study is that we did not validate the accuracy and precision of the MR systems with stimuli of different stimulus durations, especially those of shorter duration. As noted above, certain systems may not be able to reproduce auditory and visual stimuli with durations of 22.22 ms or shorter at all [24].

Finally, our study did not examine variations in stimulus duration. For example, we did not examine whether the visual stimulus (white rectangle) and the auditory stimulus (sine wave) were both presented for exactly 500 ms. Nevertheless, stimulus duration may become more consistent as the frame rate increases. The precision of stimulus duration was further investigated by Wiesing et al. who found that the combination of Unreal Engine and HTC Vive (90 Hz) achieved high accuracy and precision in stimulus duration [23].

## 5 Conclusion

In this study, we conducted quantitative tests to assess the quality of the audiovisual synchronization across many common Mixed Reality systems. Our objective was to evaluate the suitability of MR systems for research requiring accurate and precise time measurements, which is the case in neuroscience studies involving multimodal stimuli. Our findings show that no combination of HMDs (including the Meta Quest 2 and Pro, Microsoft Hololens 2, HTC Vive Pro, and Varjo XR-3), operating system, and development environment can produce good-enough audio-visual synchronization for neuroscience experiments without prior characterization and delay compensation. However, by determining the specific SOA and jitter of the auditory and visual stimuli, we were able to calibrate one of the headsets, the Varjo XR-3, to produce a consistent SOA of −3.89 ± 1.56 ms.

Summarizing, our study reveals that the audiovisual synchronization performance of MR systems can significantly vary depending on the choice of headset, operating system, and development environment. Therefore, previous research involving audiovisual stimuli using commercially available VR or AR headsets may need to be re-evaluated. Fortunately, in most cases researchers can compensate for this lag through software once they have characterized the presentation hardware. We have demonstrated here how to do that using a low-cost and custom device that is easy to set up, allowing for the replication and extension of our tests to other MR systems.
